# Transcription Factor RUNX3 Mediates Plasticity of ThGM Cells Toward Th1 Phenotype

**DOI:** 10.3389/fimmu.2022.912583

**Published:** 2022-07-04

**Authors:** Javad Rasouli, Giacomo Casella, Weifeng Zhang, Dan Xiao, Gaurav Kumar, Paolo Fortina, Guang-Xian Zhang, Bogoljub Ciric, Abdolmohamad Rostami

**Affiliations:** ^1^ Department of Neurology, Thomas Jefferson University, Philadelphia, PA, United States; ^2^ Sidney Kimmel Cancer Center, Department of Cancer Biology, Thomas Jefferson University, Philadelphia, PA, United States; ^3^ Department of Translation and Precision Medicine, Sapienza University, Rome, Italy

**Keywords:** ThGM, GM-CSF, RUNX3, T helper plasticity, neuroinflammation

## Abstract

GM-CSF-producing T helper (Th) cells play a crucial role in the pathogenesis of autoimmune diseases such as multiple sclerosis (MS). Recent studies have identified a distinct population of GM-CSF-producing Th cells, named ThGM cells, that also express cytokines TNF, IL-2, and IL-3, but lack expression of master transcription factors (TF) and signature cytokines of commonly recognized Th cell lineages. ThGM cells are highly encephalitogenic in a mouse model of MS, experimental autoimmune encephalomyelitis (EAE). Similar to Th17 cells, in response to IL-12, ThGM cells upregulate expression of T-bet and IFN-γ and switch their phenotype to Th1. Here we show that in addition to T-bet, TF RUNX3 also contributes to the Th1 switch of ThGM cells. T-bet-deficient ThGM cells in the CNS of mice with EAE had low expression of RUNX3, and knockdown of RUNX3 expression in ThGM cells abrogated the Th1-inducing effect of IL-12. Comparison of ThGM and Th1 cell transcriptomes showed that ThGM cells expressed a set of TFs known to inhibit the development of other Th lineages. Lack of expression of lineage-specific cytokines and TFs by ThGM cells, together with expression of TFs that inhibit the development of other Th lineages, suggests that ThGM cells are a non-polarized subset of Th cells with lineage characteristics.

## Introduction

Granulocyte macrophage-colony stimulating factor (GM-CSF) is a pro-inflammatory cytokine that can be expressed by both immune and tissue-resident cells (1–9). Among immune cells, T helper (Th) cells are the most abundant source of GM-CSF (2, 10). Recent studies have revealed the crucial role of GM-CSF-producing Th cells in autoimmune diseases, including in multiple sclerosis (MS) (11, 12). It is widely accepted that in MS, and in its animal model, experimental autoimmune encephalomyelitis (EAE), myelin-specific Th cells infiltrate into the CNS and initiate inflammation by acting on myeloid cells. Infiltrated Th cells are reactivated by CNS antigen-presenting cells (APCs), in particular, by dendritic cells (DCs) (13–16), and produce pro-inflammatory cytokines such as GM-CSF, which licenses the inflammatory phenotype of monocytes and monocyte-derived cells. These inflammatory monocytic cells produce reactive oxygen species (ROS) and reactive nitrogen species (RNS) that damage oligodendrocytes and neurons, leading to demyelination, neuronal loss and neurologic deficit (17, 18).

GM-CSF is expressed by several subsets of Th cells, including the newly characterized subset known as ThGM cells. ThGM cells lack expression of Th lineage-specific cytokines and master transcription factors (TFs) (19, 20). ThGM cells have been studied only minimally in health and disease, although their elevated numbers have been reported in several autoimmune diseases, including MS (6, 10, 21, 22). It has been shown that ThGM cells are highly enriched in the cerebrospinal fluid of MS patients (23). MS patients have increased frequencies of ThGM cells in their peripheral blood, and immunomodulatory therapies, such as IFN-β and dimethyl fumarate, normalize their numbers (6, 10). In mice, ThGM cells can be highly encephalitogenic in an adoptive EAE model, and they can be readily identified in the periphery and CNS of mice with EAE (19). ThGM cells, similar to Th17 cells, have plastic phenotype; in the CNS of mice with EAE they upregulate T-bet and IFN-γ, and acquire Th1-like phenotype, which notably augments their encephalitogenicity, as T-bet-deficient ThGM cells have low capacity to induce EAE (19, 24, 25).

Although it has been well established that ThGM cells adopt the Th1 phenotype in the CNS of mice with EAE, the mechanism whereby this phenotype switch occurs has not been elucidated. Here, we show that IL-12 induces IFN-γ expression in ThGM cells *via* upregulation of T-bet, both *in vivo* and *in vitro*. Adoptively transferred myelin-specific ThGM cells upregulated RUNX3 in the CNS of recipient mice in a T-bet-dependent manner. Knockdown of RUNX3 expression in ThGM cells, abrogated the phenotype switch upon stimulation with IL-12. We also compared the transcriptomes of both human and mouse ThGM cells with Th1 cells and identified a set of TFs predominantly expressed by ThGM cells that possibly directs the development of their unique phenotype.

## Results

### IL-12 Induces ThGM to Th1 Phenotype Switch *In Vitro* and in EAE

**Figure 1:**
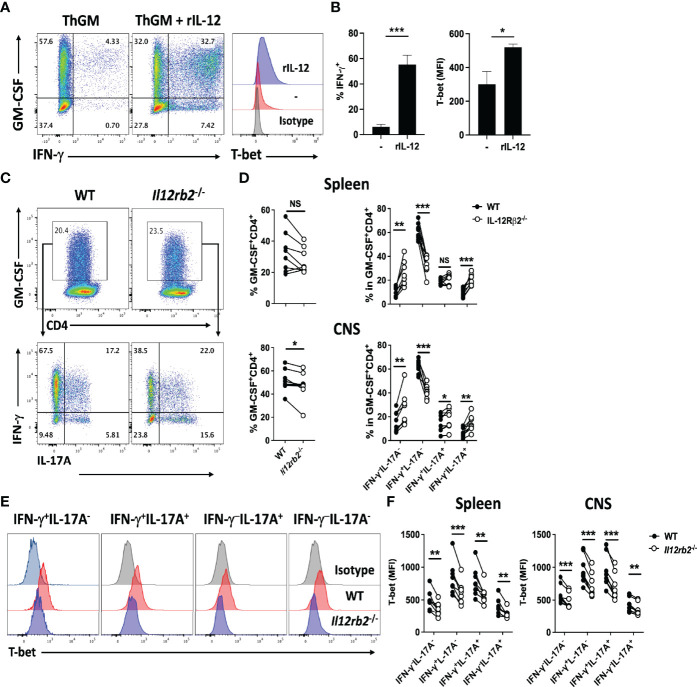
IL-12 induces T-bet expression in ThGM cells. WT naïve CD4^+^ T cells were differentiated into ThGM cells *in vitro* and treated with IL-12 in the second stimulation. **(A)** Representative flow cytometry dot plots showing GM-CSF, IFN-γ, and T-bet expression by ThGM cells after IL-12 treatment. **(B)** Proportions (%) of Th1-like cells after IL-12 treatment (left graph). Mean fluorescent intensity (MFI) of T-bet expression after IL-12 treatment (right graph). **(C)** WT (CD45.1) and *Il12rb2* ^-/-^ (CD45.2) CD4^+^ T cells were transferred into *Rag1* ^-/-^ mice (n = 8). Recipient mice were immunized for EAE induction, and cells obtained from the spleen and CNS were analyzed at the peak of disease. Representative flow cytometry dot plots showing GM-CSF, IFN-γ, and IL-17A expression by WT and *Il12rb2* ^-/-^ CD4^+^ T cells in the spleen of mice with EAE. **(D)** Proportions (%) of GM-CSF^+^ cells among WT and *Il12rb2* ^-/-^ CD4^+^ T cells analyzed in the spleen and CNS mice with EAE. Proportions (%) of different Th lineages in WT and *Il12rb2* ^-/-^ GM-CSF^+^CD4^+^ T cells analyzed in **(D). (E)** Representative histograms showing T-bet expression by different Th lineages in WT and *Il12rb2* ^-/-^ GM-CSF^+^CD4^+^ in the CNS. **(F)** MFI for T-bet expression by different Th lineages in WT and *Il12rb2* ^-/-^ GM-CSF^+^CD4^+^ in the spleen and CNS. Data shown are mean ± SEM. P-values were calculated using unpaired Student’s t-test **(A, B)** and paired Student’s t-test with Bonferroni’s multiple comparison **(C–F)**; *p < 0.05, **p < 0.01, ***p < 0.001, NS, not significant.

We have shown that ThGM cells acquire Th1 phenotype in the CNS of mice with EAE (19). To investigate a potential role of IL-12 in this phenotype switch, we first differentiated naive CD4^+^ T cells to ThGM cells with IL-1β and blocking antibodies against IFN-γ and IL-4. ThGM cells were then re-activated in the presence of IL-12. IL-12 induced notable IFN-γ and T-bet expression in ThGM cells, compared to ThGM cells reactivated without addition of IL-12, which expressed less T-bet and little IFN-γ (**Figures 1A, B**). Next, we tested whether IL-12 induces T-bet expression in ThGM cells *in vivo*. CD4^+^ T cells from WT and *Il12rb2* ^-/-^ mice were transferred into *Rag1* ^-/-^ mice, which were then immunized for EAE induction. At the peak of disease, proportions of GM-CSF^+^ WT and *Il12rb2* ^-/-^ CD4^+^ T cells in the spleen were similar (**Figures 1C, D**), while the frequencies of GM-CSF^+^ *Il12rb2* ^-/-^ cells in the CNS were somewhat reduced compared to WT cells (**Figure 1D**). The percentages of GM-CSF^+^ *Il12rb2* ^-/-^ Th1 cells were reduced in both the spleen and CNS, whereas the frequencies of *Il12rb2* ^-/-^ ThGM (GM-CSF^+^IFN-γ^-^IL-17A^-^) cells (and Th17 cells) were increased compared to WT T cells (**Figures 1C, E**). The reduction in T-bet expression by *Il12rb2* ^-/-^ Th cells was not limited to a particular subset, as all GM-CSF^+^ Th cells had reduced numbers of T-bet^+^ cells (**Figures 1E, F**). These results show that IL-12 signaling induces Th1 phenotype in ThGM cells by upregulation of T-bet expression, both *in vitro* and *in vivo*.

### T-Bet-Induced RUNX3 Is Required for Phenotype Switch From ThGM to Th1

**Figure 2:**
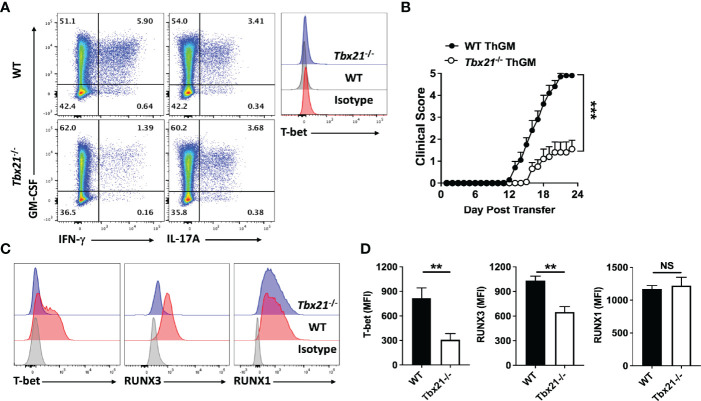
ThGM cells upregulate RUNX3 in the CNS of mice with adoptive EAE in a T-bet-dependent manner. WT and *Tbx21* ^-/-^ 2D2 naïve CD4^+^ T cells were differentiated into ThGM cells and transferred into *Rag1* ^-/-^ mice (n = 10 mice/group). **(A)** Representative flow cytometry dot plots showing GM-CSF, IFN-γ, IL-17A, and T-bet expression by WT and *Tbx21* ^-/-^ ThGM cells before adoptive transfer. **(B)** Clinical severity score of *Rag1* ^-/-^ mice with adoptive EAE. **(C)** Representative histogram of T-bet, RUNX3, and RUNX1 expression by WT and *Tbx21* ^-/-^ ThGM cells from the CNS of *Rag1* ^-/-^ mice with adoptive EAE. **(D)** MFI for T-bet, RUNX1, and RUNX3 in WT and *Tbx21* ^-/-^ ThGM cells from the CNS of *Rag1* ^-/-^ mice with adoptive EAE. Data shown are mean ± SEM. For EAE, p-values were calculated using two-way ANOVA with Bonferroni’s multiple comparison correction. Parametric datasets were analyzed using unpaired Student’s t-test with Bonferroni’s correction; **p < 0.01, ***p < 0.001, NS, not significant.

**Figure 3:**
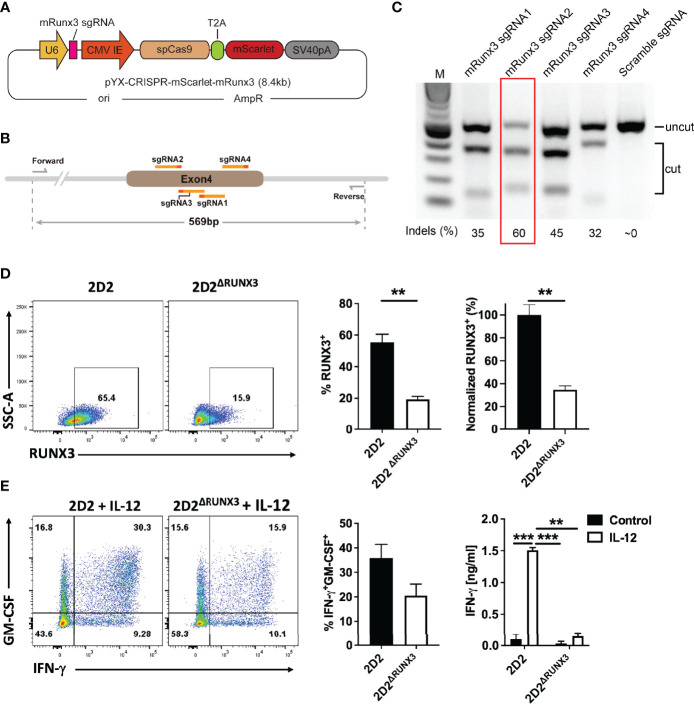
ThGM cells require RUNX3 for their plasticity toward Th1-like phenotype. **(A)** Schematic showing structure of plasmid that carries mRunx3 sgRNA, Cas9, and mScarlet under CMV promoter. **(B)** Schematic showing targets of sgRNAs and detection primers on the genomic DNA of mRunx3. **(C)** N2A-Cas9 cell line was transfected with each mRunx3 sgRNA expressing plasmids; the efficiency of knockouts was analyzed by T7E1 assay. **(D)** RUNX3 expression in ThGM cells was knocked out using CRISPR/Cas9 and remaining levels of RUNX3 in ThGM^ΔRUNX3^ cells were determined by flow cytometry. **(E)** ThGM and ThGM^ΔRUNX3^ cells were cultured with IL-12 in the second stimulation and IFN-γ concentration in cell culture supernatants was measured by ELISA. These experiments were conducted three times with similar outcomes. Data shown are mean ± SEM. P-values were calculated using unpaired Student’s t-test with Bonferroni’s correction; **p < 0.01, ***p < 0.001.

It has been shown that IL-12 induces Th1 phenotype switch in Th17 cells *via* induction of expression of RUNX1 and RUNX3 in a T-bet-dependent manner (26). To test whether these TFs play a similar role in Th1 phenotype switch of ThGM cells, we determined their levels in myelin-specific Th cells in adoptive EAE. ThGM cells were differentiated from naïve CD4^+^ T cells of 2D2 and 2D2/*Tbx21* ^-/-^ mice and transferred into *Rag1^-/-^* mice. 2D2 and 2D2/*Tbx21* ^-/-^ ThGM cells had similar compositions at the time of transfer, with comparable proportions of GM-CSF^+^ cells and low frequencies of IFN-γ^+^, IL-17A^+^, and T-bet^+^ cells (**Figure 2A**). Transferred 2D2 ThGM cells were highly encephalitogenic, whereas 2D2/*Tbx21* ^-/-^ ThGM cells induced markedly less severe disease (**Figure 2B**). 2D2/*Tbx21* ^-/-^ ThGM cells had decreased expression of RUNX3 compared to 2D2 ThGM cells, whereas RUNX1 expression was not reduced by the absence of T-bet (**Figures 2C, D**). To test the hypothesis that RUNX3 induces IFN-γ expression in ThGM cells, we knocked out RUNX3 expression in 2D2 ThGM cells by using CRISPR/Cas9 (**Figures 3A–C**), reducing the proportion of RUNX3^+^ cells by approximately 70% (**Figure 3D**). This resulted in a 50% decrease in numbers of IFN-γ^+^GM-CSF^+^ Th cells upon IL-12 stimulation, and notably reduced IFN-γ concentrations in cell culture media (**Figure 3E**). Taken as a whole, these data show that T-bet induces RUNX3 expression in ThGM cells, leading to IFN-γ expression.

### ThGM Cells Have a Unique Transcriptome Profile

**Figure 4:**
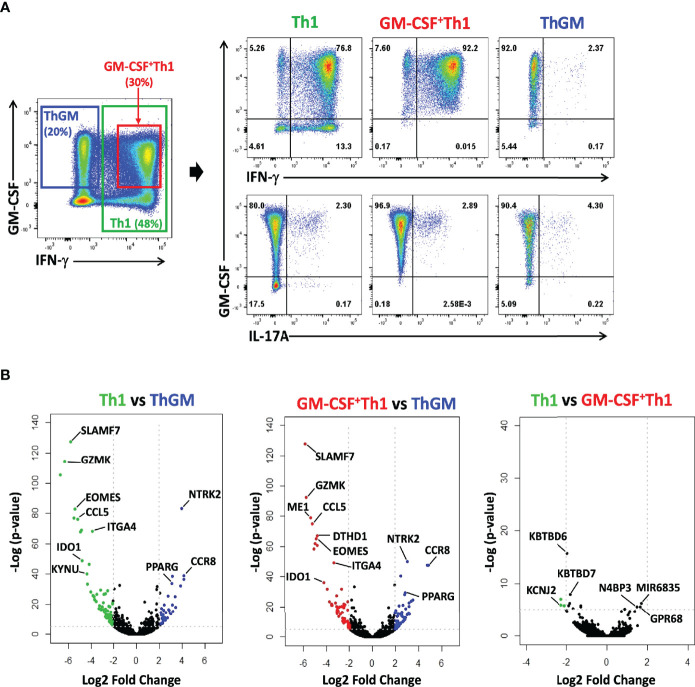
ThGM cells have a unique transcriptome profile. Human ThGM, Th1, and GM-CSF^+^Th1 cells were isolated from PB T_M_ cells using a combination of FACS sorting and cytokine capture assay (n=6 subjects). RNA was extracted and analyzed using RNA-seq analysis. **(A)** Representative flow cytometry dot plots showing GM-CSF, IFN-γ, IL-17A expression by total T_M_ cells before separation, and of isolated Th1, GM-CSF^+^ Th1, and ThGM cells isolated from PB of healthy donors. **(B)** Volcano plots showing 469 differentially expressed genes by Th1, GM-CSF^+^ Th1, and ThGM cells with 218 gense being upregulated and 251 genes downregulated by ThGM cells in comparison to both Th1 and GM-CSF^+^Th1 cells.

**Figure 5:**
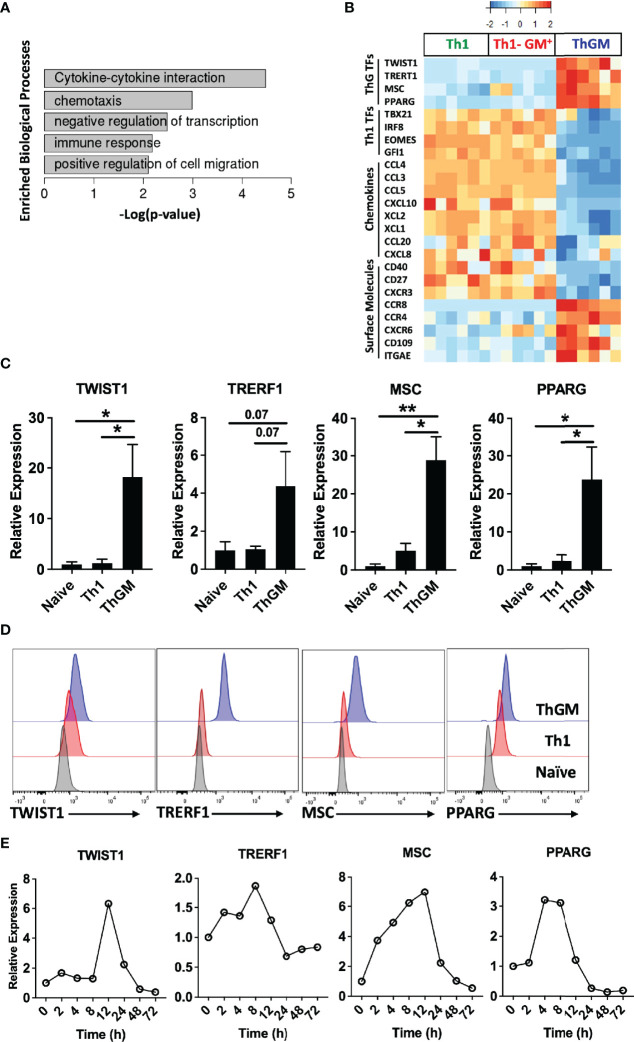
ThGM cells express a unique set of transcription factors. Human ThGM, Th1, and GM-CSF^+^Th1 (Th1-GM^+^) cells were isolated from total T_M_ cells of PB of healthy donors (n = 6 subjects) using a combination of FACS sorting and cytokine capture assay. RNA was extracted and analyzed using RNA-seq analysis. **(A)** Bar-graph showing enriched pathways for ThGM-specific genes. **(B)** Heatmap showing expression levels of ThGM- and Th1-specific TFs, chemokines, and surface molecules. **(C)** RNA expression of TWIST1, TRERF1, MSC, and PPARG by FACS-sorted naïve CD4^+^, Th1, and ThGM cells were determined by RT-PCR (n = 4 subjects). **(D)** Representative flow cytometry histograms for TWIST1, TRERF1, MSC, and PPARG expression by FACS-sorted naïve CD4^+^, Th1, and ThGM cells. **(E)** Human naïve CD4^+^ T cells were differentiated into ThGM cells and RNA was extracted at several time points. mRNA levels for TWIST1, TRERF1, MSC, and PPARG were quantified by RT-PCR. Data shown are mean ± SEM. P-values were calculated using unpaired Student’s t-test with Bonferroni’s correction; *p < 0.05, **p < 0.01.

To characterize ThGM phenotype in detail, we compared the transcriptome of ThGM cells with that of Th1 cells, in both mouse and human systems. We chose the comparison with Th1 cells because ThGM cells readily switch to Th1 cells, indicating that they are perhaps the most similar. Human natural ThGM cells (CCR4^+^CCR10^+^CXCR3^-^CXCR5^-^CD25^-^) were FACS sorted from peripheral blood memory CD4^+^ T cells (T_M_ cells; CD4^+^CD45RA^-^) of six healthy donors, and then enriched to >90% homogeneity by GM-CSF secretion assay-detection kit (**Supplementary Figure 1**). Virtually all enriched ThGM cells expressed GM-CSF, while having low expression of IFN-γ, IL-17A (**Figure 4A**), IL-4, IL-5, IL-9, and GATA3 (**Supplementary Figure 2**). Th1 cells (CCR4^-^CCR10^-^CXCR3^+^CXCR5^-^CD25^-^) were also FACS sorted, and half of Th1 cells were used to enrich GM-CSF^+^ Th1 cells using a GM-CSF secretion assay-detection kit (**Supplementary Figure 1**). We next compared the transcriptomes of ThGM, Th1, and GM-CSF^+^ Th1 cells using RNA-seq. 469 genes were differentially expressed, with a 2-fold difference in ThGM cells with 218 genes being upregulated and 251 genes downregulated (**Figure 4B**). Among the biological pathways, five pathways were enriched in ThGM cells that are mostly involved in immune response, cell migration, and cytokine interactions (**Figure 5A**).

We previously showed that both human and mouse ThGM cells have low expression of the master TFs that direct the development of established Th lineages (19), suggesting the possibility that ThGM phenotype is shaped by TFs selectively expressed in ThGM cells. Further bioinformatics analysis identified four TFs, TWIST1, MSC, TRERF1, and PPARG enriched in ThGM cells (**Figure 5B**). We validated their differential expression by RT-PCR, flow cytometry, and determined the kinetics of their expression *in vitro* (**Figures 5C–E**). TWIST1, MSC, and PPARG have been shown to have inhibitory effects on the development of other Th lineages by blocking expression of their signature cytokines and TFs (27–31). This correlates well with negative regulation of the transcription processes enriched in ThGM cells (**Figure 5A**). ThGM cells had low expression of chemokines, including CCL3, CCL4, CCL5, CCL20, CXCL8, CXCL10, XCL1, and XCL2 (**Figure 5B**), but higher expression of CXCR6, a known marker for the pathogenic Th cell (32). ThGM cells had some features of tissue resident memory T cells, including higher expression of CXCR6 and CD103 and lower expression of CD27 (**Figures 5B**). Low expression of CD27 indicates that ThGM cells are terminally differentiated (33). These data show that human ThGM cells have a unique transcriptome and that they express a specific set of TFs to a greater extent than Th1 cells.

**Figure 6:**
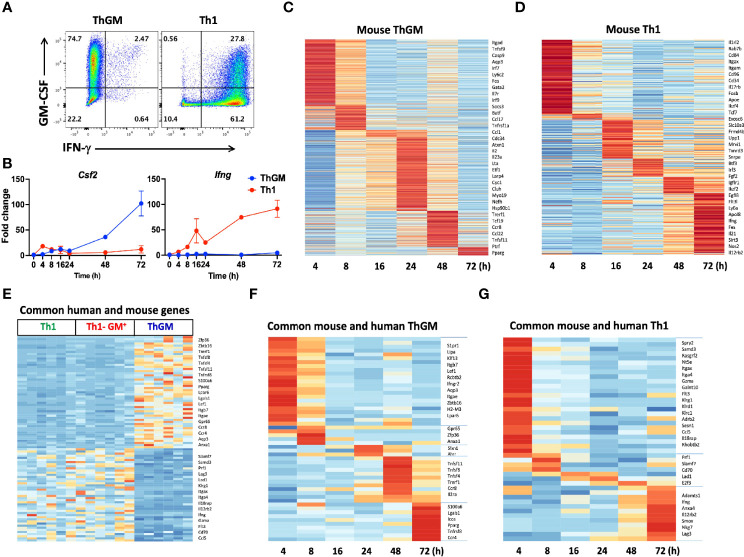
Mouse and human ThGM cells have similar transcriptomes. Mouse 2D2 naïve CD4^+^ T cells were differentiated into ThGM and Th1 cells, their RNA was extracted at different time points and analyzed by RNA-seq. **(A)** Representaive flow cytometry plots showing GM-CSF and IFN-γ expression by mouse ThGM and Th1 cells. **(B)** FPKM at different time points were normalized relative to FPKM of naive CD4^+^ T cells (0 h). Graphs showing fold change expression of GM-CSF and IFN-γ by Th1 and ThGM cells. Heatmaps for ThGM **(C)** and Th1 **(D)** -specific genes at different time points. **(E)** Differentially expressed genes by mouse and human ThGM and Th1 cells were compared. Heatmap showing expression of commonly expressed genes between *in vitro*-polarized mouse Th1 and ThGM, and natural human ThGM, GM-CSF^+^Th1, and Th1 cells. **(F)** Heatmap showing expression of commonly expressed genes between *in vitro*-polarized mouse and natural human ThGM and **(G)** Th1 cells. Data shown are mean ± SEM.

To characterize the mouse ThGM transcriptome, we differentiated naïve CD4^+^ T cells from 2D2 mice into ThGM and Th1 cells (**Figure 6A**) and collected their RNA at several time points (4-72 h) post- activation. We identified 954 genes that were upregulated in ThGM cells at different time points, while 358 genes were significantly more expressed by Th1 cells (**Figures 6C, D**). Unlike in the human system, in which ThGM and Th1 cells had similar levels of GM-CSF expression, mouse ThGM cells had significantly higher expression of GM-CSF than Th1 cells (**Figures 6A, B**). Next, we compared transcriptomes of human and mouse ThGM cells in order to identify commonly expressed genes between them. We compared human ThGM-specific genes with those selectively expressed in mouse ThGM cells at different time points. Mouse and human ThGM cells both had high expression of PPARG, TRERF1, LEF1, CCR4, CCR8, LPAR6, ITGAE, ITGB7, TNFSF11, TNFSF8, and TNFSF4 (**Figures 6E–G**).

## Discussion

Our results suggest that ThGM cells require IL-12/T-bet/RUNX3 axis for switching to Th1-like cells (ex-ThGM cells) and becoming encephalitogenic. ThGM cells, similar to Th17 cells (24, 25), upregulate T-bet and IFN-γ expression and acquire Th1-like phenotype in the CNS of mice with EAE. Given that IFN-γ plays an overall suppressive role in the development of EAE (34, 35), it is unlikely that IFN-γ substantially contributes to the encephalitogenicity of ex-ThGM cells. In the parallel case with Th17 cells (and Th1 cells as well), it has been confirmed that IFN-γ is not required for their typical encephalitogenicity (36), whereas T-bet is (37). *Tbx21* ^-/-^ ThGM cells could be less encephalitogenic than WT ThGM cells simply because of their poor survival upon transfer into recipient mice. However, a fairly large population of transferred *Tbx21* ^-/-^ ThGM cells persisted in the spleen of recipient mice, suggesting that their survival is not affected by T-bet deficiency. In addition, transferred *Tbx21* ^-/-^ ThGM cells re-isolated from the spleen expressed high levels of GM-CSF, demonstrating that they are fully viable and responsive (19). It is therefore unlikely that low encephalitogenicity of *Tbx21* ^-/-^ ThGM cells could be explained by their low survival rate. Furthermore, unlike adoptively transferred WT ThGM cells, which infiltrated the CNS of recipient mice in appreciable numbers, *Tbx21* ^-/-^ ThGM cells were largely absent from the CNS (19). These findings show that *Tbx21* ^-/-^ ThGM cells fail to accumulate in the CNS, and it remains unknown, which feature(s), induced by T-bet expression, endows ex-ThGM and ex-Th17 cells with enhanced encephalitogenicity compared to their ThGM and Th17 precursors.

Ex-ThGM cells had high expression of RUNX1 and RUNX3 in the CNS of mice with adoptive EAE, but only expression of RUNX3 was T-bet-dependent. RUNX3 has been shown to interact directly with T-bet to induce IFN-γ expression in Th1 cells (38). RUNX3 was also required for IFN-γ expression in ex-ThGM cells, as its knockout precluded IFN-γ expression in these cells even in the presence of T-bet. On the other hand, Th17 cells require both RUNX1 and RUNX3 to become encephalitogenic ex-Th17 cells (26). RUNX1 plays a crucial role in the differentiation of Th17 cells by enhancing the expression and transcriptional activity of RORγt (39). Enrichment of RUNX1 in GM-CSF-producing Th cells and its role in promoting GM-CSF expression by binding to the GM-CSF promoter and the inducible enhancer (40, 41) suggest that RUNX1 may contribute to GM-CSF production by ThGM cells rather than their plasticity. This premise is strengthened by the fact that the absence of T-bet did not alter the expression of RUNX1 and GM-CSF in ThGM cells, whereas lack of T-bet precluded RUNX3 expression and transition of *Tbx21^-/-^* ThGM cells to ex-ThGM phenotype.

We also show that IL-12 induces T-bet and RUNX3 expression in ThGM cells. However, upregulation of T-bet in ThGM cells *in vivo* is not fully dependent on IL-12 signaling, as a substantial portion of *Il12rb2* ^-/-^ ThGM cells expressed T-bet, indicating that other signals also induce T-bet expression. In addition to IL-12, other cytokines such as IL-7, IL-23, IFN-γ, and IL-18 can also induce IFN-γ expression in Th cells (24, 42–46). Even though both IFN-γ and IL-18 are known to induce IFN-γ expression in T cells (45, 46), they did not have a significant effect on the plasticity of ThGM cells *in vitro* (19). Although using IL-7 to polarize both human and mouse ThGM cells induced a great portion of IFN-γ^+^ Th cells in them (19), studying the role of IL-7 signaling on ThGM plasticity *in vivo* can be challenging due to the prominent role of IL-7 in survival and proliferation of T cells (47–49). IL-23 is another cytokine that has been shown to induce Th1-like phenotype in Th17 cells (1). Additionally, a recent study using GM-CSF fate reporter mice showed that the lack of IL-23 signaling in GM-CSF-producing Th cells reduced the number of GM-CSF^+^IFN-γ^+^ Th cells in the CNS of mice with EAE (50). We previously showed that IL-23 did not induce Th1 phenotype in either human or mouse ThGM cells *in vitro*, which could have been due to low expression of IL-23 receptors by ThGM cells compared to Th17 cells (19). Even though IL-23 did not upregulate IFN-γ in expression in ThGM cells *in vitro*, it would be worth determining whether IL-23 has an effect on GM-CSF expression and plasticity of ThGM cells *in vivo*.

Our RNA-seq analyses of human and mouse ThGM *versus* Th1 cells have failed to identify a unique TF in ThGM cells that could be considered a candidate for the master TF that directs the development of ThGM cells. However, we found a set of TFs overexpressed in ThGM cells compared to Th1 cells and two of them (PPARG and TRERF1) were differentially expressed by both mouse and human ThGM cells. Based on the known functions of these four TFs in CD4^+^ T cells, which is blocking Th1, Th2, Th17, iTreg, and Tfh cell development (28–31, 51), we propose that ThGM cells are *de facto* a “non-polarized” lineage. This lack of polarization to a particular Th lineage is likely a result of insufficient strength of polarizing signals required to induce a particular Th phenotype (e.g. Th1) in recently activated naïve CD4^+^ T cells. In this view, it is plausible that no single master TF directs the development of ThGM phenotype; it develops automatically when extracellular polarizing signals (e.g. cytokines) fail to initiate a particular polarizing program in CD4^+^ T cells with sufficient intensity to override the effects of TFs that maintain “non-polarized” phenotype of ThGM cells. IL-6 and IL-7 in the human system, and IL-1β in the mouse system, can potentially be viewed as ThGM-polarizing cytokines, as they increase proportions of GM-CSF^+^ ThGM cells. However, it is more likely that they simply enhance GM-CSF expression (19), as they also do in other lineages, but are not in themselves ThGM-polarizing signals. GM-CSF is not a permanent trait of Th cells, and ThGM cells, like other Th cells, eventually cease GM-CSF expression. These “GM-CSF^-^ ThGM” cells continued to express other cytokines such as IL-2, IL-3, and TNF. This is similar to GM-CSF expression by other Th lineages, such as Th1, Th2, and Th17, in which some cells belonging to each lineage express GM-CSF, while others do not, either because they never expressed it or eventually ceased its expression (19). This concept agrees with a recent study with GM-CSF fate reporter mice, showing a high similarity of the epigenetic landscape of GM-CSF-producing and ex-GM-CSF T_M_ cells in the CNS of EAE mice. These cells differed only in GM-CSF expression while sharing a highly similar transcriptome (50).

In other words, insufficiently potent polarizing signals (e.g. IL-12, IL-4, TGF-β) received by recently activated naïve CD4^+^ T cells fail to induce expression of polarizing master TFs (e.g. T-bet, GATA3, FoxP3) in them, leading to the development of non-polarized ThGM phenotype. However, this does not mean that the phenotype of ThGM cells is not distinct, stable, or that ThGM cells do not behave as a lineage. This concept is a departure from the current view that all Th cells are polarized to acquire specialized functions, such as fighting a particular class of pathogens (e.g. Th1 for viruses). It introduces a concept of non-specialized “generic” Th cells, which upon re-activation secrete large quantities of basic inflammatory mediators, GM-CSF, IL-2, and TNF, boosting in that way overall immunity against pathogens. Hence, even though ThGM cells do not produce cytokines specialized for clearance of a particular type of pathogen (e.g. IFN-γ), this does not mean that they are functionally irrelevant, as their capacity to induce EAE demonstrates.

The concept of non-polarized Th cells is not necessarily new. The term Th0 cells has been in use since 1989 (52) but its meaning has been arbitrary. It was initially introduced to designate Th cell clones not conforming to definitions of Th1 and Th2 cells. The Th0 designation has also been used for Th cells that develop *in vitro* in non-polarizing conditions, even if a substantial portion of them were IFN-γ-producing Th1 cells. Alternatively, Th0 cells were considered to be recently activated, immature, effector CD4^+^ T cells with a transient phenotype that within a day or so progresses into either Th1 or Th2 phenotype. However, recent findings are consistent with the view that ThGM cells are Th0 cells but with stable phenotype, at least to the extent that the phenotype of Th17 cells is stable. Several important questions about ThGM cells and their relationship with Th1 cells *in vivo* remain unanswered: 1. Should GM-CSF^-^ Th cells with overall phenotype similar to GM-CSF^+^ ThGM cell phenotype be viewed as GM-CSF^-^ ThGM cells? GM-CSF is not a Th lineage-specific cytokine, and its expression is not permanent, as most Th cells that express it eventually stop its expression (19). Hence, it is possible that some “ThGM-like” cells never expressed it, or stopped expressing it, without other major changes in their phenotype; 2. What portion of Th1 cells *in vivo* originate from ThGM cells that at some point switched their phenotype? Findings suggest that the majority of Th1 cells in the CNS of mice with EAE are ex-Th17 cells (24). It is therefore possible that in certain contexts a substantial portion of Th1 cells originate from ThGM cells; 3. How can we rigorously identify ThGM cells *ex vivo*, as a portion of them could be cells of other Th lineages that stopped expression of lineage-specific markers, such as IFN-γ; 4. What is the typical role of ThGM cells in immunity? Our knowledge thus far suggests that they are not specialized for a particular type of pathogen, but rather enhance immune responses in general by abundant expression of GM-CSF, TNF, IL-2 and FASL, which, together, potently activate APCs and induce IL-1β secretion from them (53).

In summary, our results show that ThGM plasticity toward Th1-like phenotype is required for their pathogenicity. IL-12 in a T-bet-dependent manner induces RUNX3 expression in ThGM cells resulting in their transition to Th1-like phenotype. ThGM cells have unique transcriptome, and their TF profile suggests that ThGM cells are a non-polarized Th lineage with a relatively stable phenotype.

## Materials and Methods 

### Mice and *Rag1* ^-/-^ EAE Induction

CD45.1, 2D2, *Tbx21* ^-/-^, *Il12rb2* ^-/-^ and *Rag1* ^-/-^ mice were purchased from Jackson Laboratory (Bar Harbor, ME, USA). 2D2/*Tbx21* ^-/-^ mice were generated by crossing 2D2 mice with *Tbx21* ^-/-^ mice and used for adoptive EAE experiments. All experimental procedures were performed with the approval of the Institutional Animal Care and Use Committee of Thomas Jefferson University.

To study the role of *Il12rb2* ^-/-^ in ThGM development, total CD4^+^ T cells from WT and *Il12rb2* ^-/-^ mice were purified using a CD4 isolation kit (Miltenyi Biotec). Three days before immunization, 1x10^7^ CD4^+^ T cells from WT and *Il12rb2* ^-/-^ mice at a 1:1 ratio were transferred to *Rag1* ^-/-^ recipient mice. *Rag1* ^-/-^ mice were immunized by subcutaneous injection of 200 μg MOG_35-55_ (Genscript, CA, USA) in CFA. Mice received 200 ng of pertussis toxin (Sigma-Aldrich) on days 0 and 2 p.i. and were scored daily for clinical signs as follows: 0, no sign of clinical disease; 1, paresis of the tail; 2, paresis of one hind limb; 3, paresis of both hind limbs; 4, paresis of the abdomen; 5, moribund/death.

### Mouse Th Differentiation

Naïve (CD62L^hi^CD44^-^CD25^-^CD4^+^) T cells from 2D2 mice were FACS sorted and differentiated into ThGM cells as previously described (19). Briefly, naïve T cells were cultured at a ratio of 1:4 with T cell-depleted splenocytes at a density of 1x10^6^ cell/ml. Naïve T cells were activated with MOG_35-55_ peptide (25 μg/ml) for 72 h in different Th differentiation conditions. ThGM: IL-1β (10 ng/ml), anti-IFN-γ (10 μg/ml), anti-IL-12 (10 μg/ml), anti-IL-4 (5 μg/ml) antibodies. Th1: IL-12 (20 ng/ml). For the second stimulation ThGM cells were reactivated with anti-CD3/28 (2 μg/ml) in the presence or absence of IL-12 (20 ng/ml).

### Adoptive Transfer EAE

To perform the adoptive transfer, 2D2 and 2D2/*Tbx21* ^-/-^ naïve CD4^+^ T cells were activated and differentiated into ThGM cells as described above. CD4^+^ T cells were purified using a CD4 isolation kit after the second stimulation (Miltenyi Biotec), and 1x10^7^ cells were intravenously transferred to *Rag1* ^-/-^ mice and were scored daily for clinical signs. Mice were sacrificed at disease peak (day 20 post transfer) and mononuclear cells in the CNS and spleen were analyzed by flow cytometry.

### Isolation of CNS Mononuclear Cells

CNS mononuclear cells were isolated as previously described (54). In brief, *Rag1* ^-/-^ mice with adoptive EAE were anesthetized and perfused with ice-cold PBS, and brains and spinal cords were collected. The CNS was digested in Liberase (Sigma-Aldrich) for 30 min at 37°C, then mechanically dissociated and mononuclear cells were isolated using Percoll gradient (GE Healthcare).

### Flow Cytometry and Intracellular Staining

For intracellular cytokine staining, cells isolated either from EAE mice or culture were activated with 50 ng/ml Phorbol 12-myristate 13-acetate (PMA) (Sigma-Aldrich), 500 ng/ml ionomycin (Sigma-Aldrich), and 1 μg/ml of GolgiPlug (BD Biosciences) for 4 h. Cells were washed and stained with surface antibodies (**Supplementary Table 1**). Cells were washed, fixed and permeabilized with Caltag Fix/Perm reagents (Invitrogen) following the manufacturer’s instructions. Cells were then stained with intracellular antibodies as listed in **Supplementary Table 1**.

Human samples, similar to mouse, were activated with PMA/Ionmycin/GolgiPlug, stained with surface and intracellular antibodies (**Supplementary Table 2**). Data were acquired on a FACSAria Fusion (BD Biosciences) and analyzed using FlowJo software (TreeStar).

### RNA-Seq Analysis

To perform bulk RNA-seq on human ThGM cells, total CD4^+^ T cells were purified from PBMCs using negative-selection CD4 isolation kit (Miltenyi Biotec). T_M_ cells were then purified from total CD4^+^ T cells with negative-selection CD45RA microbeads (Miltenyi Biotec) according to the manufacturer’s instructions. T_M_ cells were stained for CD4, CD45RA, CD25, CXCR5, CXCR3, and CCR4 (**Supplementary Table 2**). ThGM (CD25^-^CXCR5^-^CXCR3^-^CCR4^+^CCR10^+^) and Th1 cells (CD25^-^CXCR5^-^CXCR3^+^CCR4^-^CCR10^-^) were FACS sorted. GM-CSF^+^-ThGM and -Th1 cells were enriched based on their GM-CSF expression using GM-CSF secretion assay kit (Miltenyi Biotec) according to the manufacturer’s instructions (**Supplementary Figure 1**). Duplicate samples from three donors were FACS sorted and RNA from three cell types (ThGM, Th1, and GM-CSF^+^IFN-γ^+^ CD4^+^ T cells) were extracted using RNeasy Plus Micro kit (Qiagen).

Bulk RNA-seq analysis was performed on mouse ThGM cells that developed *in vitro*. 2D2 naïve CD4^+^ T cells were differentiated into ThGM and Th1 cells as described above. CD4^+^ T cells were sorted from several time points (0, 4, 8, 16, 24, 48, and 72 h). Biological replicates were used for three out of six time points (0, 16, and 72 h). RNA from each time point was extracted using RNeasy Plus Mini kit (Qiagen) according to the manufacturer’s instructions.

100 ng of total RNA from mouse or human samples was used to prepare libraries using TruSeq Stranded Total RNA kit (Illumina, CA, USA) following the manufacturer’s protocol. The final libraries at the concentration of 4 nM were sequenced on NextSeq 500 using 75bp paired-end chemistry. Raw FASTQ sequencing reads were mapped against the reference genome of either *Homo Sapiens* Ensembl version GRCh38 or *Mus musculus* Ensembl version GRCm38 utilizing further information from the gene transfer format (.gtf) annotation from GENCODE version GRCh38.p12 (for human) and GRCh38.p12 (for mouse) using RSEM. Total read counts, and normalized Transcripts Per Million (TPM) were obtained using RSEM’s calculate-expression function. Beforehand, differential expression, batch effects or sample heterogeneity were tested using iSeqQC (https://github.com/gkumar09/iSeqQC). Differential gene expression was tested using the DESeq2 package in R/Bioconductor. Genes were considered differentially expressed (DE) if they had adjusted p ≤ 0.05 and absolute fold change ≥ 2. All the plots were constructed using R/Bioconductor.

### RT-PCR

RNA was extracted from mouse and human T cells using RNeasy Plus Mini Kit (Qiagen). cDNA was then converted, and PCR was performed using the following FAM conjugated primer‐probe mixtures (Applied Biosystems): Trerf1 (Hs00363301), Twist1 (Hs01675818), Msc (Hs00231955), and Pparg (Hs1115513). Values were normalized to VIC conjugated GAPDH (Hs02786624) and compared to control samples.

### CRISPR/Cas9 Mediated RUNX3 Knockout

pYX-Asc plasmid was purchased from Dharmacon. pmaxGFP plasmid was purchased from Lonza. lentiCRISPR v2 was a gift from Feng Zhang (Addgene plasmid # 52961) and AAV pCAG-FLEX-mScarlet-WPRE was a gift from Rylan Larsen (Addgene plasmid # 99280). U6-sgRNA backbone-EFS-Cas9-P2A-Puromycin cassette was subcloned from LentiCRISPR v2 to pYX-Asc plasmid. P2A-Puromycin cassette was replaced by T2A-mScarlet amplified from pCAG-FLEX-mScarlet-WPRE. EFS promoter was replaced by CMV-IE promoter amplified from pmaxGFP plasmid. SV40pA was inserted after mScarlet. The acquired plasmid was named pYX-CRISPR-mScarlet.

Four sgRNAs targeting mouse Runx3 genes were designed by Benchling and synthesized from IDT (Integrated DNA Technologies, Inc.) (**Supplementary Table 3**). sgRNA oligos were annealed at room temperature and inserted into pYX-CRISPR-mScarlet to obtain mRunx3 sgRNA expression plasmids. To detect the cleavage efficiency of mRunx3 sgRNAs, mRunx3 sgRNA expression plasmids were transfected into the N2A-Cas9 cell line (Genecopoeia) separately by using lipofectamine 2000 (Invitrogen). Cells were collected and genomic DNA was extracted 24 h after transfection. A 569bp fragment flanking the sgRNAs binding sites was amplified by PCR using detection primers, and then PCR products were subjected to denaturation and reannealing using a thermocycler, purified using a Monarch PCR & DNA cleanup kit (NEB, Ipswich, MA, USA) and digested by T7E1 (T7 Endonuclease 1). Reactions were resolved using 2% TAE agarose gel electrophoresis. To determine the editing efficiency Each band was quantified with ImageJ (NIH). To perform genome editing, naïve CD4^+^ T cells were differentiated into ThGM cells for 48 h and transfected with mRunx3 sgRNA2 (5 μg per 1X10^6^ cells) with Neon transfection system (ThermoFisher Scientific) according to the manufacturer’s instructions. 24 h later, mScarlet+ ThGM cells were FACS sorted and reactivated in the presence of IL-12 (20 ng/ml) for another 48 h. RUNX3 expression was quantified by flow cytometry and cell culture supernatant was collected for cytokine quantification.

### Statistical Analysis

Statistical analysis was performed by GraphPad Prism 9 software. EAE clinical scores were analyzed using Two-way ANOVA. The paired, two-tailed student t-test was used to analyze transferred WT and knockout T cells within the same recipient mouse. The paired, two-tailed student t-test also was used to analyzed human samples after treatment with cytokines. Parametric data were analyzed using an unpaired, two-tailed Student’s t-test. The Bonferroni correction was applied for adjustment of the significance values for multiple comparisons; adjusted p ≤ 0.05 was considered significant. Data represent mean ± SEM.

## Data Availability Statement

Data related to both human and mouse RNA-seq analysis have been deposited in the NCBI Gene Expression Omnibus database with GEO accession number GSE205763.

## Ethics Statement

The studies involving human participants were reviewed and approved by Thomas Jefferson Univeirty. The patients/participants provided their written informed consent to participate in this study. The animal study was reviewed and approved by Thomas Jefferson University.

## Author Contributions

JR designed and performed experiments, evaluated and interpreted the data, and wrote the manuscript. GC performed experiments and edited the manuscript. WZ and DX, designed and executed CRISPR/Cas9 experiments, and edited the manuscript. GK performed RNA-seq analysis and data interpretation. PF and G-XZ revised the manuscript. BC supervised the study, designed the experiments, interpreted the data, and wrote the manuscript. AR supervised and financed the studies. All authors contributed to the article and approved the submitted version.

## Funding

This study was supported by the NIH (1R01AI155974-01A1) to AR.

## Conflict of Interest

The authors declare that the research was conducted in the absence of any commercial or financial relationships that could be construed as a potential conflict of interest.

## Publisher’s Note

All claims expressed in this article are solely those of the authors and do not necessarily represent those of their affiliated organizations, or those of the publisher, the editors and the reviewers. Any product that may be evaluated in this article, or claim that may be made by its manufacturer, is not guaranteed or endorsed by the publisher.

## Supplementary Material

Supplementary Figure 1 Sorting strategies for isolation of ThGM, Th1, and Th1-GM^+^ cells. Total CD4^+^ T cells were purified from PBMCs using negative-selection CD4 isolation kit. T_M_ cells were then purified from total CD4^+^ T cells with negative-selection CD45RA microbeads. T_M_ cells were stained for CD4, CD45RA, CD25, CXCR5, CXCR3, and CCR4. ThGM (CD25^-^CXCR5^-^CXCR3^-^CCR4^+^CCR10^+^) and Th1 (CD25^-^CXCR5^-^CXCR3^+^CCR4^-^CCR10^-^) cells were FACS sorted. ThGM and GM-CSF^+^ Th1 cells were enriched based on their GM-CSF expression using GM-CSF secretion assay kit.

Supplementary Figure 2 Sorted human ThGM cells do not express Th1, Th2, and Th9 markers. T_M_, ThGM, and Th1 cells were FACS-sorted, activated with PMA/Ionomycin/GolgiPlug and analyzed by flow cytometry. Representative flow cytometry dot plots showing GM-CSF, IFN-γ, IL-4, IL-5, IL-9, IL-13, and GATA3 expression by T_M_, ThGM and Th1 cells.

Supplementary Table 1 Mouse flow cytometry antibodies.

Supplementary Table 2 Human flow cytometry antibodies.

Supplementary Table 3 Sequences of sgRNAs and detection primers.
