# Supplementary material for: Transcription Factor RUNX3 Mediates Plasticity of ThGM Cells Toward Th1 Phenotype
Source: Front Immunol. 2022 Jul 4;13:912583. doi: 10.3389/fimmu.2022.912583 (PMC9289370; doi:10.3389/fimmu.2022.912583)
Supplement: Supplementary Figure 1 — Sorting strategies for isolation of ThGM, Th1, and Th1-GM+ cells. Total CD4+ T cells were purified from PBMCs using negative-selection CD4 isolation kit. TM cells were then purified from total CD4+ T cells with negative-selection CD45RA microbeads. TM cells were stained for CD4, CD45RA, CD25, CXCR5, CXCR3, and CCR4. ThGM (CD25-CXCR5-CXCR3-CCR4+CCR10+) and Th1 (CD25-CXCR5-CXCR3+CCR4-CCR10-) cells were FACS sorted. ThGM and GM-CSF+ Th1 cells were enriched based on their GM-CSF expression using GM-CSF secretion assay kit. [file DataSheet_1.docx]

**Transcription factor RUNX3 mediates plasticity of ThGM cells toward Th1 phenotype.**

Javad Rasouli^1^, Giacomo Casella^1^, Weifeng Zhang^1^, Dan Xiao^1^, Gaurav Kumar^2^, Paolo Fortina^2,3^, Guang-Xian Zhang^1^, Bogoljub Ciric^1^, Abdolmohamad Rostami^1*^.

^1^Department of Neurology, Thomas Jefferson University, Philadelphia, PA, USA

^2^Sidney Kimmel Cancer Center, Department of Cancer Biology, Thomas Jefferson University, Philadelphia, PA, USA.

^3^Department of Translation and Precision Medicine, Sapienza University, Rome, Italy

* Corresponding author

A.M. Rostami, MD, PhD, [a.m.rostami@jefferson.edu](mailto:a.m.rostami@jefferson.edu)

Department of Neurology, Jefferson Hospital for Neuroscience, Thomas Jefferson University, 900 Walnut Street, Suite 300, Philadelphia PA. 19107

**SUPPLEMENTARY FIGURES**

**Supplementary Figure 1. Sorting strategies for isolation of ThGM, Th1, and Th1-GM^+^ cells.** Total CD4^+^ T cells were purified from PBMCs using negative-selection CD4 isolation kit. T_M_ cells were then purified from total CD4^+^ T cells with negative-selection CD45RA microbeads. T_M_ cells were stained for CD4, CD45RA, CD25, CXCR5, CXCR3, and CCR4. ThGM (CD25^-^CXCR5^-^CXCR3^-^CCR4^+^CCR10^+^) and Th1 (CD25^-^CXCR5^-^CXCR3^+^CCR4^-^CCR10^-^) cells were FACS sorted. ThGM and GM-CSF^+^ Th1 cells were enriched based on their GM-CSF expression using GM-CSF secretion assay kit.


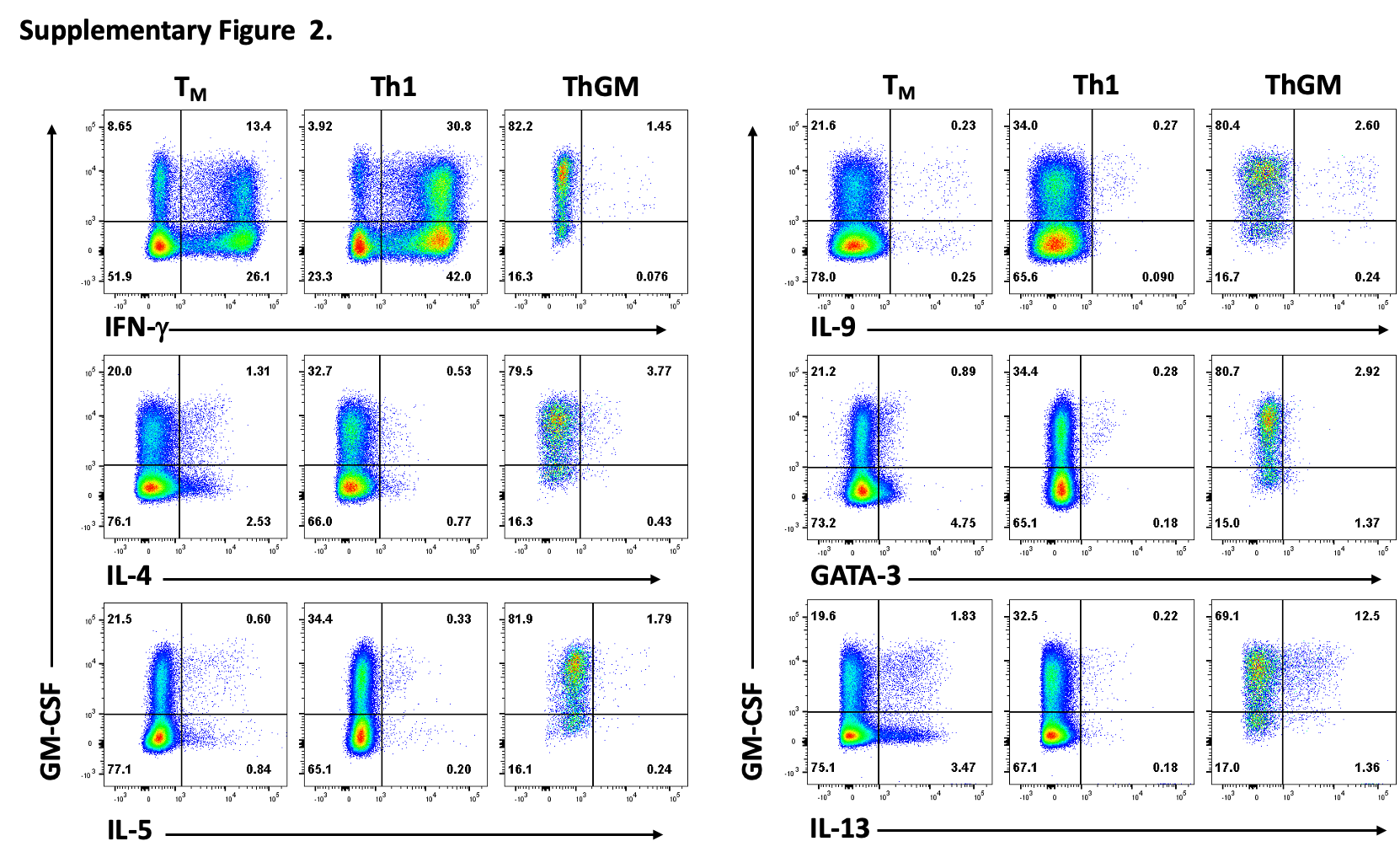


**Supplementary Figure 2. Sorted human ThGM cells do not express Th1, Th2, and Th9 markers.** T_M_, ThGM, and Th1 cells were FACS-sorted, activated with PMA/Ionomycin/GolgiPlug and analyzed by flow cytometry. Representative flow cytometry dot plots showing GM-CSF, IFN-γ, IL-4, IL-5, IL-9, IL-13, and GATA3 expression by T_M_, ThGM and Th1 cells.

**Supplementary Table 1. Mouse flow cytometry antibodies.**

| **Antigen** | **Fluorochrome** | **Clone** | **Supplier** | **Category** |
| --- | --- | --- | --- | --- |
| CD4 | Brilliant Violet 785 | RM4-5 | Biolegend | Surface |
| CD44 | DAPI | IM7 | Biolegend | Surface |
| CD45 | DAPI | 30-F11 | Biolegend | Surface |
| CD45.1 | PE-CF594 | A20 | BD Biosciences | Surface |
| CD45.2 | Brilliant Violet 650 | 104 | Biolegend | Surface |
| GM-CSF | PE | MP1-22E9 | Biolegend | Intracellular |
| IL-17A | Brilliant Violet 510 | TC11-18H10.1 | Biolegend | Intracellular |
| IFN-γ | APC | XMG1.2 | Biolegend | Intracellular |
| T-bet | PerCP-Cy5.5 | eBio4B10 | eBioscience | Intracellular |
| RUNX1 | PE | RXDMC | Invitrogen | Intracellular |
| RUNX3 | APC | 527327 | R&D Systems | Intracellular |

**Supplementary Table 2. Human flow cytometry antibodies.**

| **Antigen** | **Fluorochrome** | **Clone** | **Supplier** | **Category** |
| --- | --- | --- | --- | --- |
| CD3 | PE-Cy5 | HIT3a | Biolegend | Surface |
| CD4 | Pacific Blue | RPA-T4 | BD Biosciences | Surface |
| CD45RA | APC-Cy7 | HI100 | Biolegend | Surface |
| CD45RO | Brilliant Violet 650 | UCHL1 | Biolegend | Surface |
| CD25 | Brilliant Violet 711 | BC96 | Biolegend | Surface |
| CCR7 | Brilliant violet 785 | G043H7 | Biolegend | Surface |
| CCR4 | Percp-Cy5.5 | L291H4 | Biolegend | Surface |
| CCR10 | APC | 1B5 | BD Biosciences | Surface |
| CXCR3 | Brilliant Violet 650 | G025H7 | Biolegend | Surface |
| CXCR5 | Brilliant Violet 605 | J252D4 | Biolegend | Surface |
| GM-CSF | PE | BVD2-21C11 | BD Biosciences | Intracellular |
| IL-17A | Brilliant Violet 786 | N49-653 | BD Biosciences | Intracellular |
| IL-17A | FITC | eBio64DEC17 | eBioscience | Intracellular |
| IFN-γ | APC | 4SB3 | Invitrogen | Intracellular |
| IFN-γ | Brilliant Violet 510 | B27 | BD Biosciences | Intracellular |
| IL-4 | PE-Cy7 | MP4-25D2 | Biolegend | Intracellular |
| IL-5 | PE | TRFK5 | BD Biosciences | Intracellular |
| IL-9 | PE-Cy7 | MH9A4 | Biolegend | Intracellular |
| IL-13 | PE-Cy7 | JES10-5A2 | Biolegend | Intracellular |
| GATA3 | PE-CF594 | L50-823 | BD Biosciences | Intracellular |
| PPARγ | Alexa Fluor 594 | Polyclonal | Bioss Antibodies | Intracellular |
| MSC | FITC | Polyclonal | LSBio | Intracellular |
| TRERF1 | FITC | Polyclonal | LSBio | Intracellular |
| TWIST1 | APC | Polyclonal | R&D Systems | Intracellular |

**Supplementary Table 3. Sequences of sgRNAs and detection primers.**

**
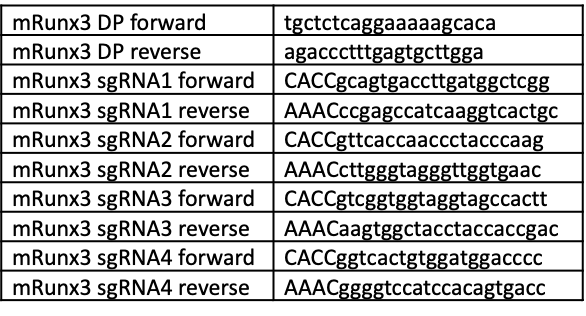
**
